# Bcl-w expression in colorectal adenocarcinoma

**DOI:** 10.1054/bjoc.1999.0897

**Published:** 1999-12-08

**Authors:** J W Wilson, M C Nostro, M Balzi, P Faraoni, F Cianchi, A Becciolini, C S Potten

**Affiliations:** CRC Epithelial Biology Laboratory, Paterson Institute for Cancer Research, Manchester, M20 4BX, UK; Laboratorio Radiobiologia, Dipartimento di Fisiopatologia Clinica, Universita Firenze, Italia

**Keywords:** Bcl-w, colorectal cancer, p53

## Abstract

We have found that the anti-apoptotic Bcl-2 family protein, Bcl-w, was frequently expressed in colorectal adenocarcinomas, with 69/75 showing positive staining with anti-Bcl-w IgG. Adenomas demonstrated a much lower frequency of Bcl-w expression (only 1 of 17), as did adenocarcinomas from other epithelial tissues such as breast (0/8), stomach (1/12) and cervix (0/12). Bcl-w status could be related to the histopathological classification of the tumours, with TNM stage III tumours showing significantly higher levels of expression than tumours of better prognostic grade (at *P* = 0.009). Those patients with node involvement also had tumours with significantly elevated levels of Bcl-w (at *P* = 0.02), compared to those which were node-negative. The results suggest that Bcl-w could play a general role in the progression from adenoma to adenocarcinoma in the colorectal epithelium. Currently, more data are being collected to allow us to assess the importance of Bcl-w for disease progression and patient survival. © 2000 Cancer Research Campaign


*British Journal of Cancer* (2000) **82**(1), 178–185
© 2000 Cancer Research Campaign
Article no. bjoc.1999.0897

### Bcl-w expression in colorectal adenocarcinoma

**JW Wilson[1], MC Nostro[1], M Balzi[2], P Faraoni[2], F Cianchi[2], A Becciolini[2] and CS Potten[1]**

[1]CRC Epithelial Biology Laboratory, Paterson Institute for Cancer Research, Manchester M20 4BX, UK;
[2]Laboratorio Radiobiologia, Dipartimento di Fisiopatologia Clinica, Universita Firenze, Italia

**Summary** We have found that the anti-apoptotic Bcl-2 family protein, Bcl-w, was frequently expressed in colorectal adenocarcinomas, with 69/75 showing positive staining with anti-Bcl-w IgG. Adenomas demonstrated a much lower frequency of Bcl-w expression (only 1 of 17), as did adenocarcinomas from other epithelial tissues such as breast (0/8), stomach (1/12) and cervix (0/12). Bcl-w status could be related to the histopathological classification of the tumours, with TNM stage III tumours showing significantly higher levels of expression than tumours of better prognostic grade (at $P = 0.009$). Those patients with node involvement also had tumours with significantly elevated levels of Bcl-w (at $P = 0.02$), compared to those which were node-negative. The results suggest that Bcl-w could play a general role in the progression from adenoma to adenocarcinoma in the colorectal epithelium. Currently, more data are being collected to allow us to assess the importance of Bcl-w for disease progression and patient survival. © 2000 Cancer Research Campaign

**Keywords:** Bcl-w; colorectal cancer; p53

*bcl-w* is a member of the *bcl-2* gene family and was initially characterized by Gibson et al (1996). *bcl-w* codes for a protein of 22 kDa apparent molecular weight. The Bcl-w protein was demonstrated to be of similar efficacy to Bcl-$x_L$ in suppressing apoptosis of haemopoietic cell lines, induced by a number of cytotoxic stimuli including γ-radiation, interleukin (IL)-3 withdrawal, dexamethasone, but not CD95 ligation. Bcl-w's role in promoting cell survival was further illustrated by the breeding of *bcl-w*-deficient mice (Ross et al, 1998). Male, homozygous, mutant mice demonstrated progressive testicular degeneration and were sterile.

The studies presented in the current manuscript suggest that expression of Bcl-w may play a role in the progression of colorectal carcinogenesis. We have previously characterized the differential expression of Bcl-2 and p53 in colorectal tumours (Watson et al, 1996), and shown an inverse relationship between Bcl-2 expression and the accumulation of p53 immunoreactivity (indicative of *p53* mutation). Bcl-2 was found to be most frequently expressed in adenomas relative to adenocarcinomas. Here we present results demonstrating the frequent expression of Bcl-w in colorectal adenocarcinomas but not in adenomas, in contrast to Bcl-2. The relationship between Bcl-w, Bcl-2 and p53 status and other clinical histopathological parameters is discussed.

### MATERIALS AND METHODS

### Patient samples

Samples were obtained from patients undergoing radical surgery for colorectal cancer. Exclusion criteria for the study were: age greater than 75 years, presence of synchronous, distant metastases or residual tumour after surgery and previous history of neoplasm.

*Received 11 February 1999*
*Revised 26 May 1999*
*Accepted 14 June 1999*

*Correspondence to:* JW Wilson

### Pathology

Tumours were classified as follows: proximal (right) tumours – from the caecum to the splenic flexure; distal (left) tumours – from the splenic flexure to the end of the sigmoid; and rectal tumours. Tumours were identified as either adenocarcinoma or mucinous adenocarcinoma. On the basis of differentiation, tumours were divided into three groups: well differentiated, moderately differentiated and poorly differentiated, according to World Health Organization classification (Jass and Sobin, 1989). All patients were staged according to Dukes' classification, as modified by Dukes and Gabriel (Gabriel et al, 1935) and according to the tumour–node–metastasis (TNM) classification (Hermanek and Sobin, 1987).

### Tumour ploidy

Tumour ploidy was assessed by flow cytometric determination of DNA content. Multiple fragments of tumour were minced with a scalpel in a citrate-buffered solution to obtain a single nuclei suspension. The suspension of the standard cell population was obtained by scraping of the mucosa, cut 10 cm above the neoplasm. Both suspensions were filtered through a 50 μm nylon mesh to remove cell clumps and the cell density adjusted to $1 \times 10^6$ cells $ml^{-1}$. Samples were stained with propidium iodide according to the method of Vindelov (Vindelov and Christensenn, 1990).

Samples of tumour, normal mucosa and mixed tumour and normal epithelial cells were analysed using a FACScan flow cytometer (Becton Dickinson) to give a single parameter

Note: Some of the data included in this study was originally presented as a poster at the joint IACR/BACR meeting, at Trinity College, Dublin, June 1998. The poster contained data from 50 cases and showed the same trends as reported here. The addition of new cases to the study has shown that the relationship between p53 and Bcl-w expression is significant, although we were unable to report this as such in the initial presentation. In the initial study we did report that tetraploid tumours had significantly elevated Bcl-w expression. Although we still see a strong association between increased ploidy and increased Bcl-w expression, we can no longer report this as being significant.

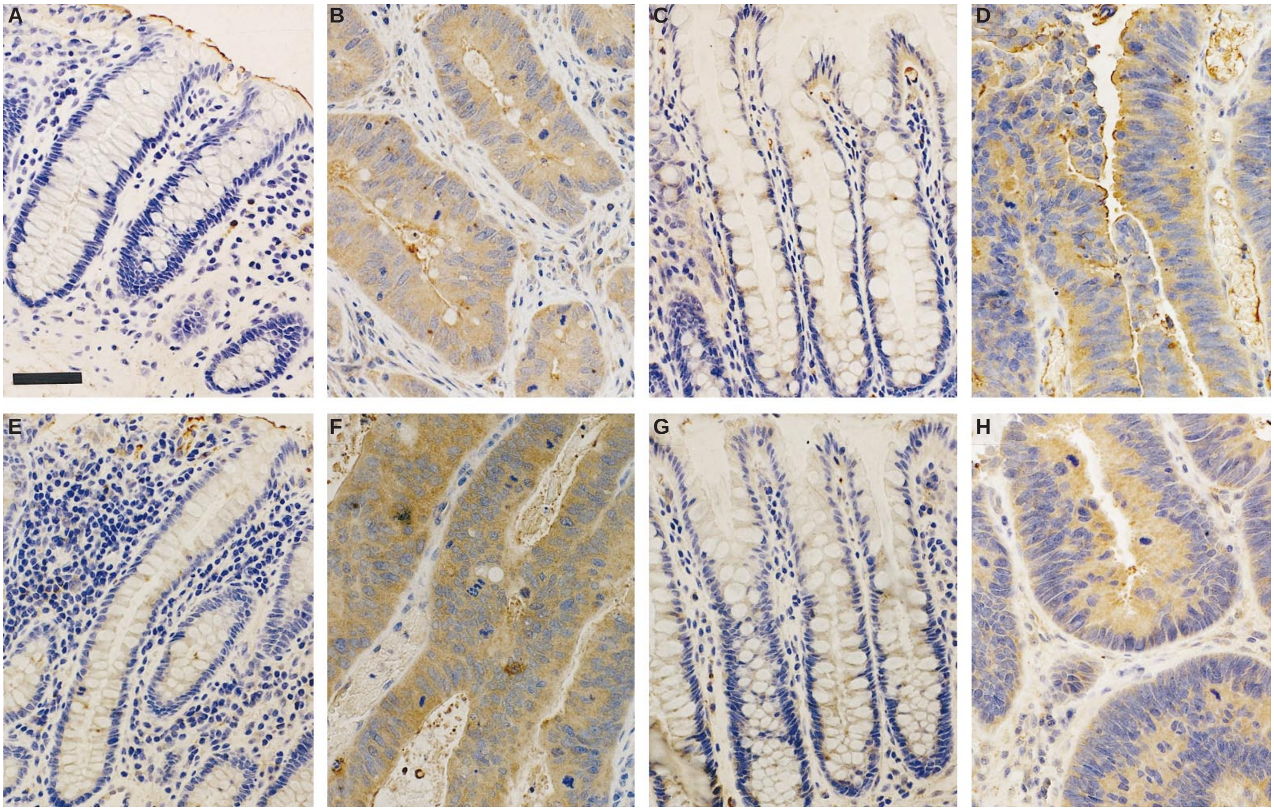

**Figure 1** Bcl-w immunoreactivity in colorectal adenocarcinomas – part I. Four pairs of normal and tumour tissue (A + B; C + D; E + F; G + H) from different patients are shown. The epithelia of the normal mucosal samples (A, C, E and G) demonstrated no significant immunoreactivity. Tumours shown are TNM stage I (Dukes' A) – D and H; TNM stage II (Dukes' B) – A; TNM stage III (Dukes' C1) – F. Bar shown in plate **A** = 50 μm

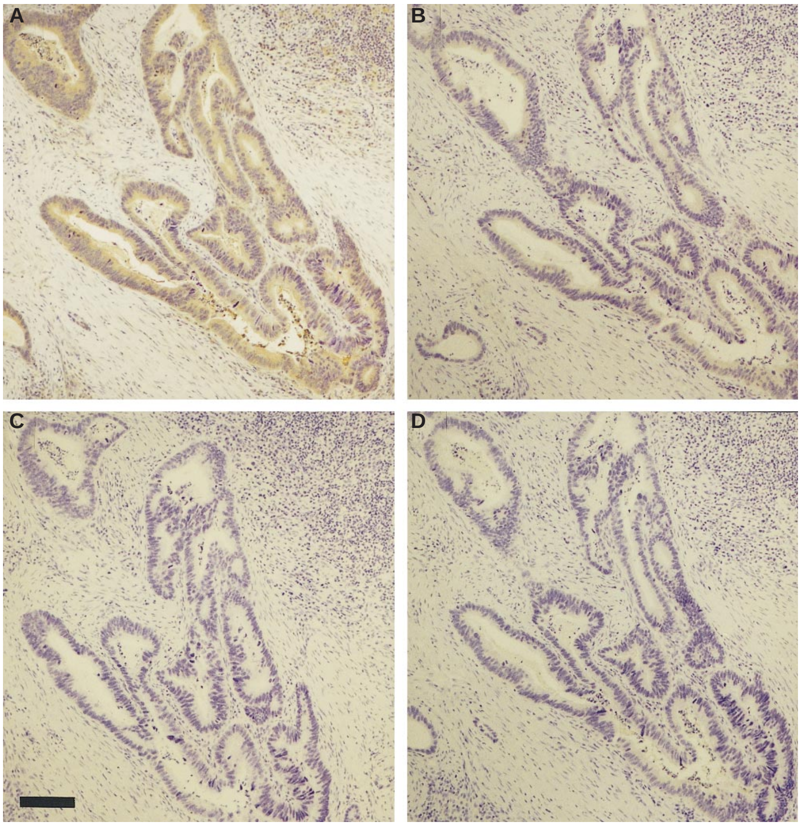

**Figure 2** Bcl-w immunoreactivity in colorectal adenocarcinomas – part II. Tumour shown in **A–D** is an adenocarcinoma, TNM stage 3 (Dukes' stage C1). Plates show tumour sections incubated with anti-Bcl-w IgG (**A**); a non-specific goat IgG – 0.67 μg $ml^{-1}$ (**B**); anti-Bcl-w IgG pre-incubated with antigenic peptide (**C**); secondary antibody alone (**D**). Bar in plate **C** = 100 μm

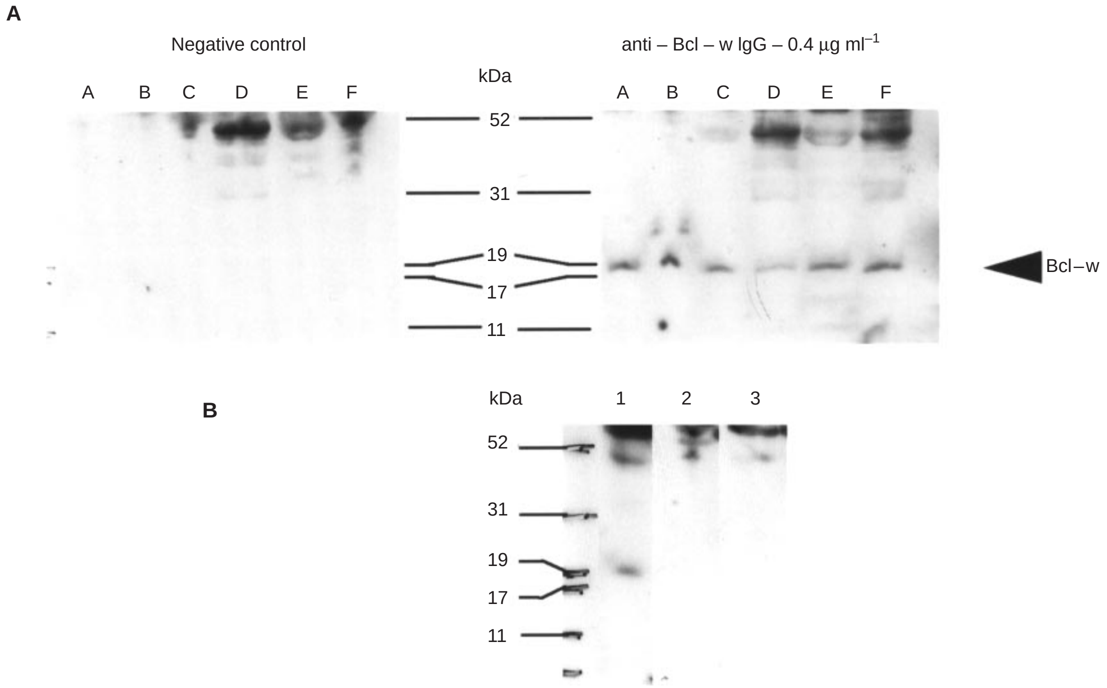

**Figure 3** (**A**). Western blot of Bcl-w immunoreactivity. Panels show membranes incubated in the absence (left), or presence (right), of primary antibody. Molecular size markers are indicated. Lanes **A, B, C, D, E** and **F** are samples prepared from six different adenocarcinomas (**A** and **F** are Dukes' A; **B** and **D** are Dukes' B; **C** and **E** and Dukes' C1). It may be noted that the secondary antibody used for Western analysis shows immunoreactivity against a c. 50 kDa size protein. Other secondary antibodies were tested and all showed similar immunoreactivity against a c. 50 kDa protein. (**B**) Immunoblots of sample F from Figure 2A, incubated with anti-Bcl-w IgG (1), secondary antibody alone (2) or with anti-Bcl-w IgG pre-incubated with excess antigenic peptide (3)

integrated fluorescence histogram. DNA ploidy was defined as the DNA index (DI), i.e. the ratio between the mean channel number of the G0/G1 peak of the tumour cells and that of the normal epithelial cells. Tumours with DI = 1 were defined as diploid and tumours with lower or higher values were considered aneuploid. Cases with DI value between 1.9 and 2.1 were classified as tetraploid tumours, if this peak represented more than 20% of the cell population. Analysis of the S phase fraction (SPF) was performed using Multicycle (Phoenix) and CellFit software (Becton Dickinson), using the Sum of Broadened Rectangles model for diploid samples and the polynomial model for aneuploid cases.

### Antibodies

### *Primary antibodies*

Goat anti-Bcl-w IgG (n19: sc6171) was obtained from Santa Cruz Biotechnology (via Autogen Bioclear, Devizes, UK). Mouse anti-Bcl-2 IgG (clone 124: M0887) and mouse anti-p53 IgG (DOH7: M7001) were obtained from Dako (High Wycombe, UK).

### *Secondary antibodies*

Rabbit anti-goat, biotin-conjugated IgG was obtained from Vector Labs (Peterborough, UK). Goat anti-mouse, biotin-conjugated IgG was obtained from Pierce and Warriner (Chester, UK).

### Immunohistochemistry

Immunohistochemistry was carried out as previously described (Wilson and Potten, 1996). Briefly, sections were dewaxed in xylene and transferred to absolute alcohol. Endogenous peroxidase activity was blocked by incubation of sections in 3% $H_2O_2$/methanol. Sections were then rehydrated through a graded series of alcohols, prior to microwave-based antigen retrieval. Sections were incubated in pre-block solution (10%, 0.2 μm filtered serum in phosphate-buffered saline (PBS)-goat serum for anti-p53 and -Bcl-2; rabbit serum for anti-Bcl-w). Incubation with primary antibodies (diluted in fresh pre-block solution) was performed overnight, at 4°C in an humidified chamber. Anti-Bcl-w IgG was used at a concentration of 0.67 μg $ml^{-1}$; anti-Bcl-2 IgG was used at a concentration of 2 μg $ml^{-1}$ and anti-p53 IgG was used at a concentration of 4 μg $ml^{-1}$. After a brief wash with TBS–Tween, sections were incubated with biotinylated secondary antibody (1:200 dilution), for 1 h at room temperature. Vector ABC reagents were used, in accordance with manufacturers' instructions, to amplify the immunoreactivity. Detection of immunoreactivity was performed using 3′3′-diaminobenzidine (DAB) as a chromogen. Slides were counterstained with thionine blue, prior to mounting with XAM permanent mount.

### Western blotting

Tumour lysates were prepared by homogenizing pieces of tumour tissue (previously frozen in liquid nitrogen and stored at –80°C) in Laemlli buffer, in the presence of protease inhibitors (Complete™, Boehringer Mannheim). The protein concentration of the samples was determined using BCA assay reagents from Pierce (Pierce and Warriner). Forty micrograms of protein (in 30 μl) was loaded onto 10% Bis–Tris Nupage gels (Novex, Oxford, UK) and run at 200 V for 35 min using MES (2-[N-morpholino] ethane sulphonic acid) running buffer (Novex). Proteins were transferred on to Hybond

 *© 2000 Cancer Research Campaign*

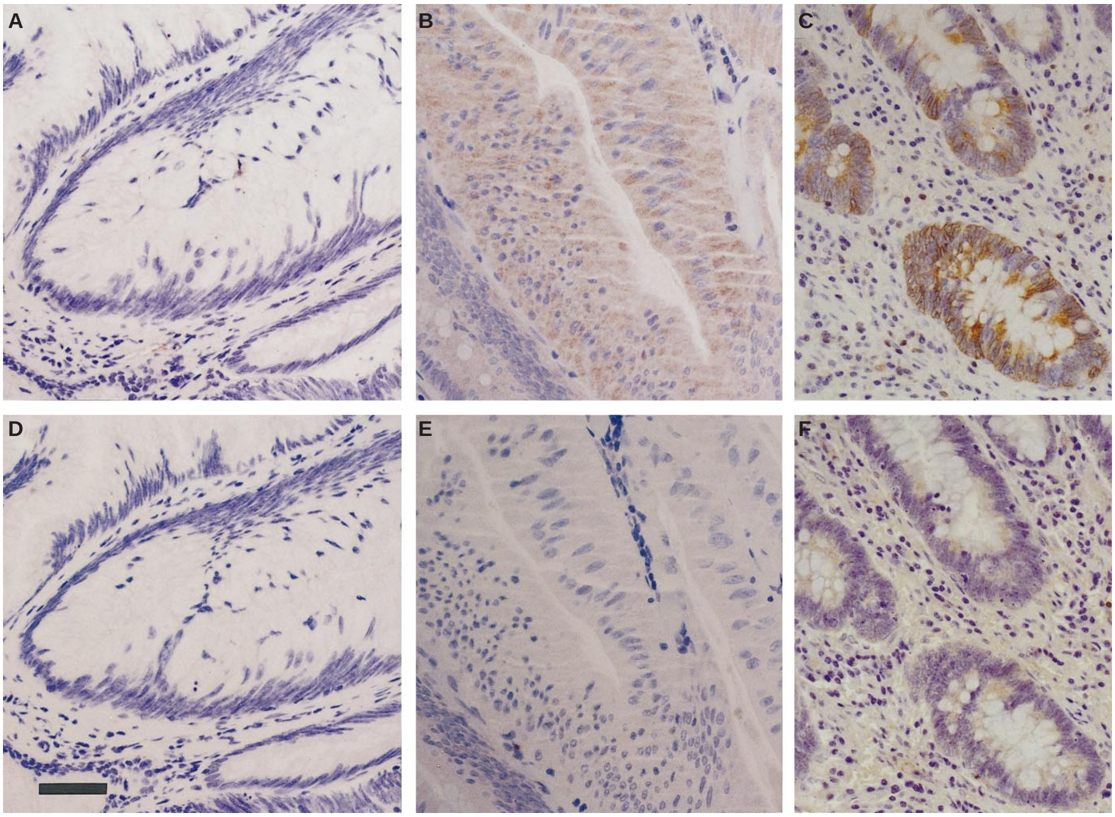

**Figure 4** Bcl-w expression in adenomas. Three different colorectal adenomas are shown. Adenoma 1 (**A** and **D**) and adenoma 2 (**B** and **E**) were incubated with anti-Bcl-w IgG (**A** and **B**) and n/s goat IgG (**D** and **E**). Adenoma 2 was the only adenoma to show significant immunoreactivity. Adenoma 3 was incubated with either anti-Bcl-2 IgG (**C**) or anti-Bcl-w IgG (**D**). The bar in plate D = 50 µm

ECL (Amersham, UK), using Tris Glycine Transfer Buffer (Novex). After transfer, the membrane was washed briefly in TBS, prior to overnight incubation in 4% dried milk/1% rabbit serum in TBS–Tween 20 (0.1%), at 4°C. The next day the membrane was incubated with anti-Bcl-w antibody (0.4 µg $ml^{-1}$ dilution) in 4% dried milk/1% rabbit serum in TBS–Tween 20 (0.1%), for 2.5 h on an orbital shaking table. The membrane was then washed (for 2 min) twice with TBS–Tween 20 (0.1%), prior to incubation with rabbit anti-goat IgG-horseradish peroxidase (human adsorbed: Santa Cruz Biotechnology/Autogen Bioclear) at 1/200 dilution in 4% milk/1% human serum in TBS–Tween (0.1%) for 1 h. The membrane was subsequently washed 4 times (12 min each wash) in TBS–Tween 20 (0.1%). Immunodetection was performed using ECL-plus reagents and ECL-hyperfilm (Amersham). To block specific immunoreactivity, primary antibody was also pre-incubated with a tenfold excess of Bcl-w peptide blocking solution (Santa Cruz/Autogen Bioclear), overnight at 4°C.

### Tumour scoring

Tumour sections were viewed by two independent observers. A consensus score out of three was given for both the intensity of immunoreactivity (INT) observed and the proportion of epithelial cells within the tumour that showed immunoreactivity (PROP). The product of the two scores was used to give a final score for the degree of immunoreactivity (total score: TS). Tumours were scored without prior knowledge of tumour histopathology.

### Statistics

Data were analysed using SPSS for Windows. Data were compared using a non-parametric Kruskal–Wallis test for analysis of variance, and a Mann–Whitney test was used to assess the difference between individual data sets. The level of significance was determined at $P \leq 0.05$.

### RESULTS

### Bcl-w expression

#### *Colorectal adenocarcinomas*

Of the 75 colorectal adenocarcinomas examined, 69 (92%) demonstrated Bcl-w immunoreactivity. Examples of staining observed in tumours from four different patient samples are shown in Figure 1. Also shown are normal mucosa samples from the same patients, taken from sites 10 cm from the tumour; these showed no significant Bcl-w immunoreactivity. The Bcl-w staining observed in the tumour samples was cytoplasmic in location and could be blocked by pre-incubation of antibody with an excess of antigenic peptide. In addition, no significant immunoreactivity was observed when tissue sections were incubated with non-specific goat immunoglobulin, at an equivalent protein concentration to the primary antibody (Figure 2).

By Western blot, a band of c.20 kDa could be detected in six out of six tumour samples examined (Figure 3A), which is in close agreement with the molecular weight of 22 kDa, previously

© 2000 Cancer Research Campaign 

**Table 1** Relationship between Bcl-w immunoreactivity and tumour pathology

| | Bcl-w INT score | Bcl-w PROP score | Bcl-w total score |
|---|---|---|---|
| Sex | (Mean values ± standard error) | | |
| Male ($n$ = 33) | 1.5 ± 0.2 | 2.1 ± 0.2 | 3.6 ± 0.5 |
| Female ($n$ = 42) | 1.5 ± 0.1 | 2.5 ± 0.1 | 4.3 ± 0.4 |
| Site | | | |
| Right ($n$ = 24) | 1.5 ± 0.2 | 2.2 ± 0.2 | 3.8 ± 0.6 |
| Left ($n$ = 23) | 1.5 ± 0.2 | 2.3 ± 2 | 4 ± 0.7 |
| Rectum ($n$ = 28) | 1.5 ± 0.2 | 2.3 ± 0.2 | 4 ± 0.5 |
| TNM stage (1 N.D.) | | | |
| I ($n$ = 16) | 1.7 ± 0.2 | 2.4 ± 0.2 | 4.5 ± 0.8 |
| II ($n$ = 34) | 1.2 ± 0.1 | 2.1 ± 0.2 | 3.1 ± 0.4 |
| III ($n$ = 24) | 1.9 ± 0.2 | 2.7 ± 0.1 | 5 ± 0.5 |
| Tumour ploidy (2 N.D.) | | | |
| Diploid ($n$ = 28) | 1.4 ± 0.2 | 2.1 ± 0.2 | 3.8 ± 0.6 |
| Near diploid ($n$ = 11) | 1.3 ± 0.2 | 2.2 ± 0.3 | 3.2 ± 0.8 |
| Tetraploid ($n$ = 8) | 1.7 ± 0.3 | 2.8 ± 0.2 | 4.8 ± 0.9 |
| Aneuploid ($n$ = 21) | 1.5 ± 0.2 | 2.4 ± 0.1 | 3.7 ± 0.6 |
| Polyploid ($n$ = 5) | 2.1 ± 0.3 | 2.4 ± 0.4 | 5.5 ± 1.4 |
| Node involvement | | | |
| 1 (zero, $n$ = 51) | 1.3 ± 0.12 | 2.1 ± 0.1 | 3.5 ± 0.4 |
| 2 (N1, $n$ = 17) | 2 ± 0.2 | 2.7 ± 0.1 | 5.4 ± 0.6 |
| 3 (N2, $n$ = 5) | 1.8 ± 0.4 | 2.6 ± 0.2 | 4.9 ± 1.3 |
| 4 (N3, $n$ = 2) | 0.8 | 3 | 2.3 |
| 5 (Any: N1+N2+N3, $n$ = 24) | 1.9 ± 0.2 | 2.7 ± 0.1 | 5 ± 0.5 |
| Histotype | | | |
| Adenocarcinoma a $n$ = 56) | 1.6 ± 1 | 2.4 ± 0.1 | 4.2 ± 0.4 |
| Mucinous expression < 50% ($n$ = 9) | 1.8 ± 0.3 | 2.4 ± 0.2 | 4.9 ± 1 |
| Mucinous ($n$ = 10) | 0.9 ± 0.2 | 1.9 ± 0.3 | 2 ± 0.5 |

reported by Gibson et al (1996). The appearance of this 20 kDa band could be blocked by the pre-incubation of anti-Bcl-w IgG with an excess of antigenic peptide (Figure 3B).

### *Colorectal adenomas*

In comparison to the adenocarcinomas, only one of 17 adenomas demonstrated Bcl-w immunoreactivity, whereas six out of nine adenomas tested were positive for Bcl-2 expression (see Figure 4).

### *Adenocarcinomas from other epithelial tissues*

As we had observed a high frequency of Bcl-w immunoreactivity in the colorectal adenocarcinomas, other adenocarcinomas from different epithelial tissues were examined. Tumour specimens were obtained for breast, cervix and stomach. Zero out of eight breast tumours and zero out of 12 cervical tumours examined were positive for Bcl-w and only one out of 12 stomach tumour demonstrated immunoreactivity (Figure 5).

### Relationship between Bcl-w expression and other factors (Table 1)

### *Sex*

There were 33 male patients (mean age (± s.e.m.) 63.3 ± 1.7) and 42 female patients (mean age 63.1 ± 1.4) included in the study. Twenty-nine out of 33 (88%) of tumours from male patients, and 40/42 (95%) of tumours from female patients demonstrated some degree of Bcl-w expression. Kruskal–Wallis/Mann–Whitney analysis revealed that the proportion of cells demonstrating expression of Bcl-w (PROP: see Materials and Methods) was significantly greater in tumours from female patients (at $P$ = 0.016). This difference was not reflected in any difference in the intensity (INT) of Bcl-w immunoreactivity, or the total score (TS; where TS = PROP score × INT score) for Bcl-w immunoreactivity, between tumours from male and female patients.

### *Site of tumour*

Tumours were classified, according to their location within the large bowel. The current data set included 24 right-sided, 23 left-sided and 28 rectal tumours. Kruskal–Wallis analysis showed there to be no relationship between Bcl-w expression and site of tumour.

### *Tumour histopathology*

***TNM stage*** By TNM staging criteria, there were 16 stage I, 35 stage II and 24 stage III tumours (one not determined). Kruskal–Wallis analysis demonstrated a relationship between TNM stage and Bcl-w staining intensity ($P$ = 0.022) and Bcl-w total score ($P$ = 0.029). Mann–Whitney analysis showed that both Bcl-w intensity and Bcl-w total score were significantly greater in stage III tumours relative to stage II tumours, both at $P$ = 0.009. Classification of tumours according to Dukes' staging showed a similar relationship to Bcl-w expression (data not shown).

***Histotype*** Tumour histotype was defined as being adenocarcinoma ($n$ = 56), adenocarcinoma with mucinous expression (less than 50%: $n$ = 9) and mucinous adenocarcinoma ($n$ = 10). There was a significant difference ($P$ = 0.046) between the Bcl-w total scores of tumours of different histotype. Mann–Whitney analysis demonstrated that the Bcl-w total scores for mucinous adenocarcinomas were significantly less than those for adenocarcinomas and adenocarcinomas with mucinous expression (less than 50%), at $P$ = 0.023 and $P$ = 0.035 respectively. This set of tumours was further analysed in relation to p53 status (see p53 and bcl-2 below).

***Grade of differentiation*** Tumours were graded as either well ($n$ = 3), moderately ($n$ = 55) or poorly differentiated ($n$ = 6); one adenocarcinoma was not classified. Mucinous adenocarcinomas ($n$ = 10) were graded as unclassified. Within the classified groups, there was no significant difference in Bcl-w status. The unclassified (mucinous) tumours had significantly lower Bcl-w INT scores ($P$ = 0.007) and Bcl-w total scores ($P$ = 0.017) than the moderately differentiated group of tumours.

### *Tumour ploidy*

Although increased ploidy was associated with increased Bcl-w expression, analysis of Bcl-w status in relation to tumour ploidy revealed no significant relationship between the two.

### *Node involvement*

There were 51 node-negative tumours (group N1 – see Table 1) and 24 tumours with varying degrees of node involvement (group N5). Kruskal–Wallis/Mann–Whitney analysis showed that Bcl-w expression was significantly greater in the node-positive group. This was the case for the proportion of tumour epithelial cells showing Bcl-w immunoreactivity ($P$ = 0.033), the intensity of Bcl-w immunoreactivity ($P$ = 0.022) and the Bcl-w total score ($P$ = 0.02).

### *Bcl-2 and p53*

p53 and Bcl-2 status were also established for the colorectal adenocarcinomas examined. p53 expression was observed with a

 © 2000 Cancer Research Campaign

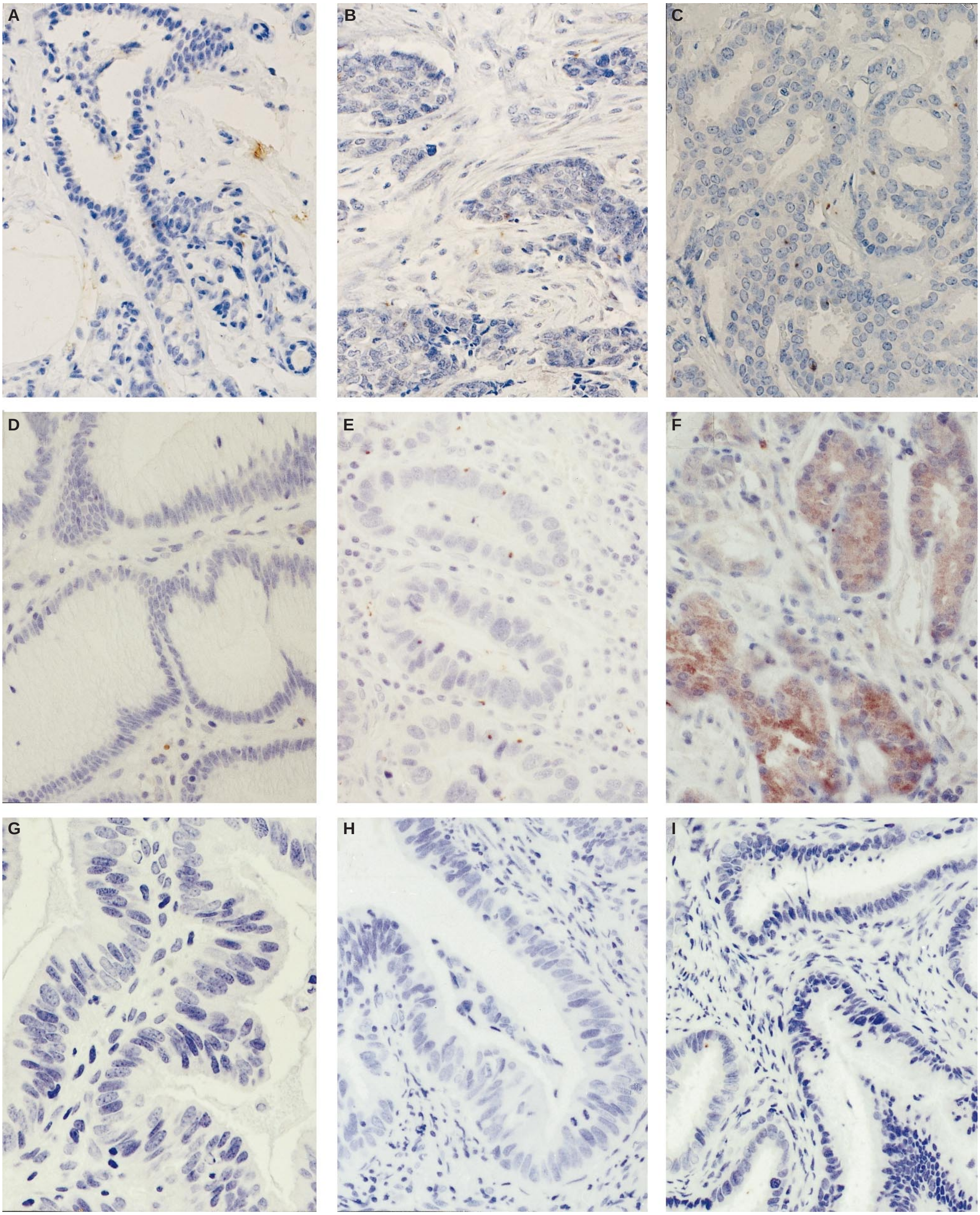

**Figure 5** Bcl-w expression in other adenocarcinomas. Breast (**A**–**C**), stomach (**D**–**F**) and cervix (**G**–**I**) adenocarcinomas. Bcl-w immunoreactivity was observed only in one stomach tumour (**F**)

similar profile to that of Bcl-w and was expressed in 76% of the adenocarcinomas. Bcl-2 was observed in only 41% of adenocarcinomas, compared to its expression in 67% of adenomas. Spearman's correlation coefficients were calculated to determine the relationship, if any, between Bcl-w status and p53 and Bcl-2 status. This revealed significant, positive correlations between the proportion of p53-immunoreactive tumour epithelial cells and (a), the proportion of Bcl-w immunoreactive tumour epithelial cells ($r$ = 0.344, $P$ = 0.002); (b), the intensity of Bcl-w staining ($r$ = 0.296, $P$ = 0.01); and (c), the total score for Bcl-w immuno-

© 2000 Cancer Research Campaign

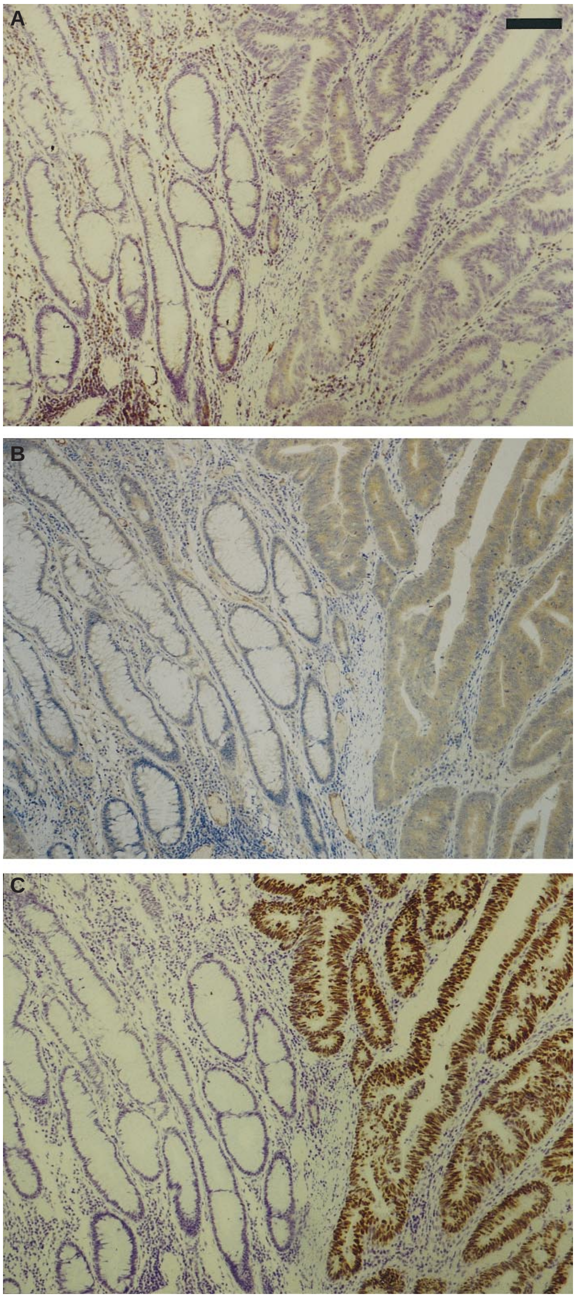

**Figure 6** Contrasting Bcl-2 (**A**) Bcl-w (**B**) and p53 (**C**) expression in a TNM stage 2 (Dukes' B) adenocarcinoma with adjacent adenomatous tissue (left). The adenocarcinoma shows strong staining for p53 and Bcl-w in contrast to the adjacent adenomatous tissue. The bar in plate A = 100 μm reactivity ($r$ = 0.327, $P$ = 0.004). There was also a significant, positive correlation between the total score for p53 immunoreactivity and the total score for Bcl-w immunoreactivity ($r$ = 0.241, $P$ = 0.037).

Some adenocarcinoma samples contained adjacent adenomatous tissue. These samples allowed excellent visualization of the contrast between the positively-stained adenocarcinoma tissue and the adenomatous tissue, which was negative for both Bcl-w and p53 immunoreactivity (Figure 5). The low level of Bcl-w expression in mucinous adenocarcinomas (as mentioned above), was correlated with a low level of p53 expression in these tumours, with Bcl-w PROP score showing strong, positive correlation with the p53 PROP score ($r$ = 0.787; $P$ = 0.007) and p53 TS ($r$ = 0.765; $P$ = 0.01).

Kruskal–Wallis analysis revealed that the proportion of tumour epithelial cells demonstrating p53 expression was significantly influenced by the site of the tumour ($P$ = 0.048). Mann–Whitney analysis showed that the proportion of tumour epithelial cells expressing p53 was greater in left-sided tumours compared to right-sided tumours ($P$ = 0.017). No significant difference was observed between right-sided and rectal tumours ($P$ = 0.063), and left-sided and rectal tumours ($P$ = 0.64)

Like Bcl-w, Bcl-2 expression was also related to TNM stage. Bcl-2 intensity was shown to be significantly different between the stages, by Kruskal–Wallis analysis ($P$ = 0.021). Mann–Whitney analysis showed that the intensity of Bcl-2 immunoreactivity was significantly greater in stage I tumours than in either stage II ($P$ = 0.01), or stage III ($P$ = 0.038) tumours. However, no significant difference was observed in Bcl-2 total score between the different groups.

### *Bcl-w as a prognostic indicator?*

We have been following up this group of patients for evidence of relapse, metastatic disease and mortality. At the time of writing, follow-up information was available for 63 of the 75 cases examined. Of the 63 cases with known status, nine patients had died: four from metastatic disease and five from local relapse. Amongst the group of surviving patients, 46 were free from disease, five had experienced metastases and three had local relapse. The mean follow-up period for patients included in this study is currently only 24 months. This is too short a period to allow proper evaluation of the relation between Bcl-w expression and future incidence of metastatic disease, relapse and death, for a disease such as colorectal cancer. However, we will continue follow-up for these patients in order to assess this.

### CONCLUSIONS

The current studies have demonstrated that Bcl-w expression was observed in greater than 90% of colorectal adenocarcinomas examined. Bcl-w was not observed in any of the normal mucosal samples studied and in only one of 17 colorectal adenomas. It was interesting to observe that adenocarcinomas from other epithelial tissues did not express Bcl-w.

Bcl-w appears to have an opposite pattern of expression to Bcl-2, which previously has been shown to be more frequently expressed in colorectal adenomas relative to adenocarcinomas (Watson et al, 1996). However, there was no significant, inverse correlation between Bcl-2 status and Bcl-w status in the adenocarcinomas examined. p53 expression did correlate well with Bcl-w expression. There were also data to suggest that increased Bcl-w expression could be related to tumour progression, with TNM stage III tumours demonstrating higher Bcl-w expression than stage II tumours. The reduced expression of Bcl-w in mucinous adenocarcinomas relative to the other tumours was a very interesting finding. As mentioned in the results, this was correlated with a low level of p53 expression. In addition, these tumours were predominantly right-sided (7/10), of TNM stage II (Dukes' B) (8/10), and showed no evidence of node involvement (9/10).

More data are clearly required to establish if Bcl-w status can be related in any way to the chemo- or radio-sensitivity of the tumour

© 2000 Cancer Research Campaign cells, clinical outcome, and patient survival. As Bcl-w has been identified as a potent suppressor of apoptosis (Gibson et al, 1996), it could confer significant survival advantage to tumour. The precise mechanism whereby Bcl-w expression is up-regulated also remains to be established. No germline mutations within the *bcl-w* gene have been reported (Gibson et al, 1996). Gibson et al (1996) demonstrated that the *bcl-w* gene was located on chromosome 14, at band q11. Although lymphoid leukaemias frequently show chromosomal aberrations at 14q11, Gibson et al thought that it would be hard to define the functional role of Bcl-w in these malignancies, due to the close linkage of the *bcl-w* gene and the *tcra* locus. The 14q11 region has been reported to show alteration in colorectal cancer cell lines by Gagos et al (1995). However, there are no current data demonstrating that a chromosomal rearrangement results in increased *bcl-w* expression. The clonogenic expansion of Bcl-2-expressing stem cells, the putative site of initial transformation events in colorectal cancer, is thought to explain the high expression of Bcl-2 in adenomas. Bcl-w on the other hand is not expressed in the normal mucosa, at least not at levels which allow detection.

Several questions need to be addressed by further studies. Is the dysregulation of Bcl-w expression a common step in the progression from adenoma to adenocarcinoma in colorectal cancer? Also, why is dysregulated Bcl-w expression specific to colorectal tumours, within the group of epithelial tumours so far examined? We are initiating further studies to assess the importance of Bcl-w in determining the apoptotic sensitivity of colorectal tumour cells and will be following up patients to more thoroughly assess the relationship between Bcl-w expression, disease progression and patient survival.

### ACKNOWLEDGEMENTS

This work was funded by the Cancer Research Campaign and grants CNR PF ACRO 96.00508.39 and MURST60%.

### Note addied in proof

Bcl-w immunohistochemistry and Western blots have since been confirmed by use of a second anti-Bclw lgG, from R&D Systems (Cat # AF 824) (Abingdon, Oxon, UK).

### REFERENCES

Gabriel WB, Dukes C and Bussey HRJ (1935) Lymphatic spread of cancer in the rectum. *Br J Surg* **23**: 39–413

Gagos S, Hopwood VL, Iliopoulos D, Kostakis A, Karayannakos P, Yatzides H, Skalkeas GD and Pathak S (1995) Chromosomal markers associated with metastasis in two colon cancer cell lines established from the same patient. *Anticancer Res* **15**: 369–378

Gibson L, Holmgreen SP, Huang DCS, Bernard O, Copeland NG, Jenkins NA, Sutherland GR, Baker E, Adams JM and Cory S (1996) *bcl-w*, a novel member of the *bcl-2* family, promotes cell survival. *Oncogene* **13**: 665–675

Hermanek P and Sobin LH (eds) (1987) *TNM Classification of Malignant Tumours*, 4th edn. Springer-Verlag: Berlin

Jass JR and Sobin LH (1989) Histological typing of intestinal tumours. In: *WHO International Histological Classifications of Tumours*, 2nd edn. Springer-Verlag: Berlin

Ross AJ, Waymire KG, Moss JE, Parlow AF, Skinner MK, Russell LD and MacGregor GR (1998) Testicular degeneration in *Bclw*-deficient mice. *Nat Genet* **18**: 251–256

Vindelov LL and Christensenn IJ (1990) A review of techniques and results obtained in one laboratory by an integrated system of methods designed for routine clinical flow cytometric analysis. *Cytometry* **11**: 753–770

Watson AJM, Merritt AJ, Jones LS, Askew JN, Anderson E, Becciolini A, Balzi M, Potten CS and Hickman JA (1996) Evidence for reciprocity of bcl-2 and p53 expression in human colorectal adenomas and carcinomas. *Br J Cancer* **73**: 889–895

Wilson JW and Potten CS (1996) Immunohistochemical localization of BAX and BAD in the normal and BCL-2 null gastrointestinal tract. *Apoptosis* **1**: 183–190

*© 2000 Cancer Research Campaign*
